# Multi-Enzymatic Cascade for Efficient Deracemization of dl-Pantolactone into d-Pantolactone

**DOI:** 10.3390/molecules28145308

**Published:** 2023-07-10

**Authors:** Lijun Jin, Xun Liu, Tairan Wang, Yi Wang, Xueting Zhou, Wangwei Mao, Yinjun Zhang, Zhao Wang, Jie Sun, Xiangxian Ying

**Affiliations:** Key Laboratory of Bioorganic Synthesis of Zhejiang Province, College of Biotechnology and Bioengineering, Zhejiang University of Technology, Hangzhou 310014, China; jinlijun130@163.com (L.J.); lx0925124X@163.com (X.L.); 13591715817@163.com (T.W.); huzaiziy@163.com (Y.W.); sadie598851321@163.com (X.Z.); yingxx22@foxmail.com (W.M.); zhangyj@zjut.edu.cn (Y.Z.); hzwangzhao@163.com (Z.W.)

**Keywords:** multi-enzymatic cascade, l-pantolactone dehydrogenase, conjugated polyketone reductase, deracemization, dl-pantolactone

## Abstract

d-pantolactone is an intermediate in the synthesis of d-pantothenic acid, which is known as vitamin B_5_. The commercial synthesis of d-pantolactone is carried out through the selective resolution of dl-pantolactone catalyzed by lactone hydrolase. In contrast to a kinetic resolution approach, the deracemization of dl-pantolactone is a simpler, greener, and more sustainable way to obtain d-pantolactone with high optical purity. Herein, an efficient three-enzyme cascade was developed for the deracemization of dl-pantolactone, using l-pantolactone dehydrogenase from *Amycolatopsis methanolica* (*Ame*LPLDH), conjugated polyketone reductase from *Zygosaccharomyces parabailii* (*Zpa*CPR), and glucose dehydrogenase from *Bacillus subtilis* (*Bs*GDH). The *Ame*LPLDH was used to catalyze the dehydrogenated l-pantolactone into ketopantolactone; the *Zpa*CPR was used to further catalyze the ketopantolactone into d-pantolactone; and glucose dehydrogenase together with glucose fulfilled the function of coenzyme regeneration. All three enzymes were co-expressed in *E. coli* strain BL21(DE3), which served as the whole-cell biocatalyst. Under optimized conditions, 36 h deracemization of 1.25 M dl-pantolactone d-pantolactone led to an *e.e.*_p_ value of 98.6%, corresponding to productivity of 107.7 g/(l·d).

## 1. Introduction

As a chiral alcohol, d-pantolactone serves as an important intermediate in the synthesis of d-pantothenic acid (vitamin B_5_), which is widely used as a pharmaceutical and cosmetic ingredient as well as a food and feed additive [1,2]. In commercial production, d-pantothenic acid is synthesized through the condensation reaction between d-pantolactone and β-alanine, and d-pantolactone is mainly prepared through the lactonase-catalyzed resolution of racemic dl-pantolactone [3,4,5,6]. In the process of enzymatic resolution, the generated enantiopure d-pantothenic acid is separated from the remaining l-pantolactone and further converted into d-pantolactone by a chemical lactonization step, requiring the consumption of energy, solvents, and acids/alkalis [7]. In efforts to develop a simpler and greener process, the oxidoreductase-based asymmetric synthesis of d-pantolactone has received increasing attention in recent years [7,8,9,10,11,12,13]. These efforts have mainly focused on the enzymatic reduction of ketopantolactone to d-pantolactone with the assistance of a glucose dehydrogenase/glucose coenzyme regeneration system. For instance, an *E. coli* strain co-expressing conjugated polyketone reductase from *Saccharomyces cerevisiae* (*Sce*CPR1) and glucose dehydrogenase from *Exiguobacterium sibiricum* (*Es*GDH) was applied for the asymmetric reduction of ketopantolactone, providing 458 mM d-pantolactone with an *e.e.*_p_ value of 99.9% and a yield of 91.6% after 6 h [8]. Ketopantolactone is prone to spontaneous hydrolysis into ketopantoic acid at neutral pH, which is unfavorable for the direct reduction of ketopantolactone to d-pantolactone. Thus, the deracemization of dl-pantolactone as a more stable substrate is more advantageous for practical purposes. More importantly, the deracemization process via an oxidation–reduction sequence allows us to transform a cheap racemate into a single enantiomer (e.g., d-pantolactone), while reducing byproduct formation and circumventing the isolation of intermediates [14,15,16,17].

In the process of enzymatic deracemization, dl-pantolactone is catalyzed by three oxidoreductases for efficient coenzyme regeneration: l-pantolactone dehydrogenase (LPLDH), ketopantolactone reductase (KPLR), and glucose dehydrogenase (GDH). Enzymatic deracemization consists of the stereoselective dehydrogenation of l-enantiomer and the subsequent asymmetric reduction of ketopantolactone to d-enantiomer. It is crucial that both l-pantolactone dehydrogenase and ketopantolactone reductase possess high activity and strict enantioselectivity. However, enzyme resources for the deracemization of dl-pantolactone are relatively limited; thus, lactonase-catalyzed kinetic resolution is still the leading approach. Specifically, known l-pantolactone dehydrogenases are membrane-bound enzymes, and ascertaining their heterologous expression remains challenging [13,18]. Mining novel enzymes or engineering known enzymes is essential to meet the requirements of activity and enantioselectivity for the efficient deracemization of dl-pantolactone [19,20].

For multi-enzymatic reactions, enzyme cascades combine the advantages of biocatalysis and one-pot multi-step reactions [21]. In the case of dl-pantolactone deracemization, FMN-dependent l-pantolactone dehydrogenases oxidize the substrate with flavin reduction, and electrons from the reduced flavin are captured by electron acceptors to regenerate the oxidized flavin. Thus, whole-cell biocatalysts outperform free enzymes in the deracemization of dl-pantolactone [22]. In addition, the use of whole-cell biocatalysts co-expressing multiple enzymes can improve the mass transfer efficiency between enzymes and the cell envelope can provide protection for intracellular enzymes [23]. In multi-enzymatic reactions, the fusion of multiple enzymes can be a viable method for generating proteins with improved catalytic activity [24,25,26]. It must be noted that, often, not every enzymatic reaction in an enzyme cascade will reach the optimal conditions, and balancing different catalytic properties is necessary to account for synergistic effects [27,28].

**Scheme 1:**
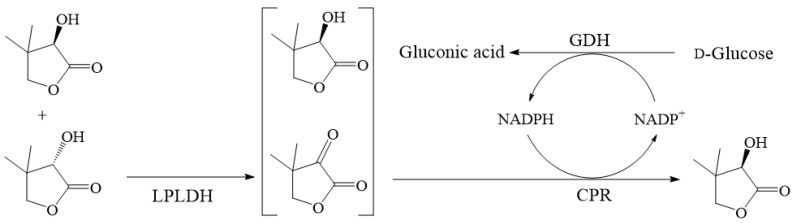
One-pot deracemization of dl-pantolactone to its d-enantiomer through a biocatalysis cascade using *E. coli* cells co-expressing l-pantolactone dehydrogenase (LPLDH), conjugated polyketone reductase (CPR), and glucose dehydrogenase (GDH).

We previously developed a three-enzyme cascade approach to mediate the one-pot, two-step reduction of (*E*/*Z*)-citral to (*S*)-citronellol, a chiral alcohol with a rose fragrance [29]. Inspired by this work, a new three-enzyme system using LPLDH, conjugated polyketone reductase (CPR), and GDH was constructed to achieve deracemization of dl-pantolactone into d-pantolactone (Scheme 1). Each enzyme involved in the process was carefully selected, and the enzymatic properties of newly mined l-pantolactone dehydrogenase and ketopantolactone reductase were characterized. In addition, genetic fusion of ketopantolactone reductase and glucose dehydrogenase was attempted to increase the catalytic efficiency. Finally, the optimization of whole-cell biotransformation enabled efficient deracemization of dl-pantolactone at high concentrations.

## 2. Results and Discussion

### 2.1. Three-Enzyme Cascade for Deracemization of dl-Pantolactone

A whole-cell biocatalyst co-expressing *Sce*CPR1 and *Es*GDH in *E. coli* was previously constructed to catalyze the asymmetric reduction of ketopantolactone to d-pantolactone. To upgrade the asymmetric reduction to the deracemization process, it is necessary to use l-pantolactone dehydrogenase with strict l-enantioselectivity to initiate the dehydrogenation of l-pantolactone and keep the d-pantolactone unreacted. l-pantolactone dehydrogenase from *Nocardia asteroides* (*Nas*LPLDH) was purified from the wild-type strain and was able to catalyze the dehydrogenation of l-pantolactone into ketopantolactone [30]. The amino acid sequence encoding *Nas*LPLDH was chosen to perform a homology search in the NCBI database. Four putative l-pantolactone dehydrogenases were selected from *Nocardia cyriacigeorgica* GUH-2 (91% sequence identity), *Nocardia farcinica* IFM 10152 (88% sequence identity), *Amycolatopsis methanolica* 239 (76% sequence identity), and *Cnuibacter physcomitrellae* strain XA(T) (71% sequence identity) and named *Ncy*LPLDH, *Nfa*LPLDH, *Ame*LPLDH, and *Cph*LPLDH, respectively. All of the genes were synthesized onto the pET28a plasmid and overexpressed in *E. coli* BL21(DE3) (Figure S1). When the substrate specificity was investigated, all five LPLDHs showed the capacity to catalyze the dehydrogenation of l-pantolactone but not d-pantolactone, indicating their strict l-enantioselectivity.

**Figure 1:**
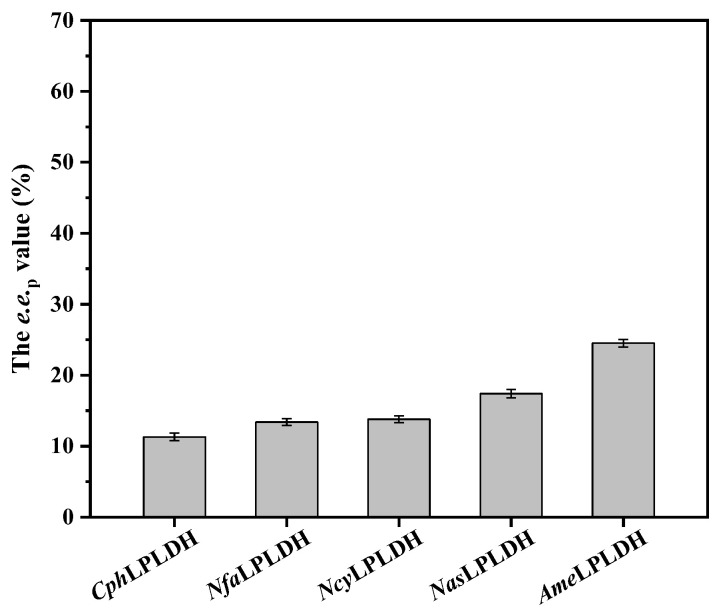
Catalytic activity screening of l-pantolactone dehydrogenases. Reaction mixture (10 mL) consisted of 200 g/L wet cells co-expressing LPLDH, SceCPR1 and EsGDH, 1 M dl-pantolactone, 1.5 M glucose and 200 mM PBS buffer (pH 7.0). Constant pH was maintained through titration with 1 M NaOH. Reaction was run at 30 °C and 600 rpm for 24 h. Data represent mean values ± SD from three independent experiments.

To further screen l-pantolactone dehydrogenase with high catalytic activity, each recombinant plasmid harboring the LPLDH-encoding gene was individually introduced into competent cells containing the pACYCDuet-1-*SceCPR1-EsGDH* plasmid, resulting in the recombinant strains co-expressing different l-pantolactone dehydrogenases, *Sce*CPR1 and *Es*GDH. Sodium dodecyl sulfate–polyacrylamide gel electrophoresis (SDS-PAGE) analysis showed good expression levels of all tested enzymes (Figure S2). As whole-cell biocatalysts, the strains co-expressing LPLDH, *Sce*CPR1 and *Es*GDH, increased the *e.e.*_p_ value from 10.5% to 24.5%, demonstrating that they were feasible for enzymatic deracemization of dl-pantolactone (Figure 1). Among them, *Ame*LPLDH had the highest *e.e.*_p_ value (24.5%) and was selected for further studies.

**Figure 2:**
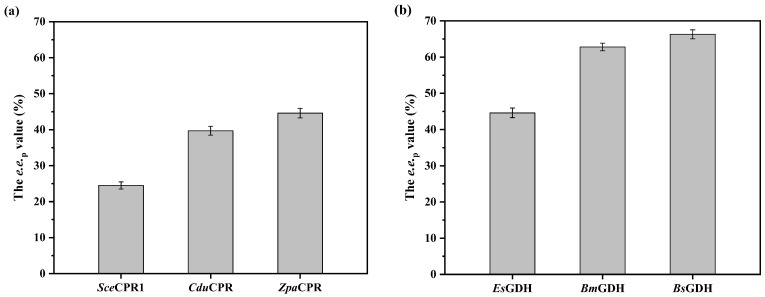
Catalytic activity screening of (**a**) conjugated polyketone reductases and (**b**) glucose dehydrogenases. Reaction mixture (10 mL) consisted of 200 g/L wet cells co-expressing LPLDH, CPR and GDH, 1 M dl-pantolactone, 1.5 M glucose, and 200 mM PBS buffer (pH 7.0). Constant pH was maintained through titration with 1 M NaOH. Reaction was run at 30 °C and 600 rpm for 24 h. Data represent mean values ± SD from three independent experiments.

Similar to l-pantolactone dehydrogenase, the high activity and strict d-enantioselectivity of conjugated polyketone reductase facilitate the efficient reduction of ketopantolactone to d-pantolactone. Unlike the limited resources of LPLDH, the number of characterized conjugated polyketone reductases rapidly increases [7,8,9,10,11,12,13]. The conjugated polyketone reductase from *Candida dubliniensis* CD36 (CduCPR) and a newly mined enzyme from *Zygosaccharomyces parabailii* (ZpaCPR) were individually co-expressed with EsGDH through the construction of recombinant plasmids pACYCDuet-1-*CduCPR-EsGDH* and pACYCDuet-1-*ZpaCPR-EsGDH*, respectively [9]. *E. coli* cells harboring these recombinant plasmids were used to catalyze the reduction of ketopantolactone, affording the single d-enantiomer. The recombinant plasmids were individually introduced into competent cells possessing pET28a-*AmeLPADH*, resulting in the recombinant strains co-expressing AmeLPLDH, conjugated polyketone reductase (SceCPR1, CduCPR or ZpaCPR), and EsGDH (Figure S3). ZpaCPR had the best catalytic performance in the deracemization of dl-pantolactone, increasing the *e.e*._p_ value from 24.5% to 44.6% (Figure 2a). In addition to LPLDH and CPR, glucose dehydrogenase, which is responsible for coenzyme regeneration, is required for the efficient deracemization of dl-pantolactone. Glucose dehydrogenases from *Bacillus megaterium* (BmGDH) and *Bacillus subtilis* (BsGDH) have been well characterized, and their catalytic performance was compared with that of EsGDH [27,31,32]. *E. coli* strains co-expressing AmeLPLDH, ZpaCPR, and different GDHs were constructed and used as whole-cell biocatalysts (Figure S4). Among the tested GDHs, BsGDH increased the *e.e.*_p_ value up to 66.3% (Figure 2b). Therefore, the subsequent whole-cell biocatalysts consisted of AmeLPLDH, ZpaCPR, and BsGDH.

### 2.2. Purification and Characterization of Key Enzymes AmeLPLDH and ZpaCPR

**Figure 3:**
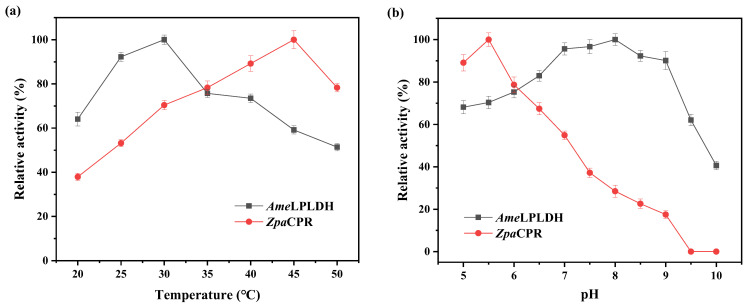
Effect of (**a**) temperature and (**b**) pH on AmeLPLDH and ZpaCPR activity. (**a**) Relative activity of 100% for AmeLPLDH and ZpaCPR represents 1.47 U/mg (assayed at 30 °C, pH 6.5) and 4.73 U/mg (assayed at 45 °C, pH 6.0). (**b**) The relative activity of 100% for AmeLPLDH and ZpaCPR represents 1.81 U/mg (assayed at 30 °C, pH 8.0) and 5.63 U/mg (assayed at 45 °C, pH 5.5). Data represent mean values ± SD from three independent experiments.

**Figure 4:**
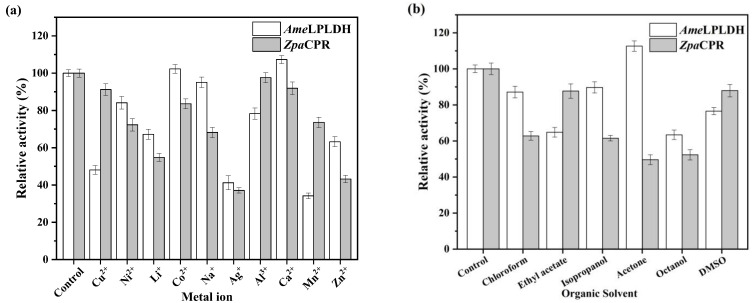
Effect of (**a**) metal ions and (**b**) organic reagents on AmeLPLDH and ZpaCPR activity. Relative activity of 100% represents 1.81 U/mg (assayed at 30 °C, pH 8.0) and 5.63 U/mg (assayed at 45 °C, pH 5.5), respectively.

As newly mined enzymes, AmeLPLDH and ZpaCPR were purified and then characterized to gain insights into their enzymatic and catalytic properties. The purification was conducted by affinity chromatography (Figure S5). Various factors, including temperature, pH, metal ions, and organic solvents, were investigated. The influence of temperature was determined over a range of 20–50 °C, and the highest activity for AmeLPLDH and ZpaCPR was observed at 30 and 45 °C, respectively (Figure 3a). To determine the effect of pH on activity, an enzyme assay was carried out at pH ranging from 5.0 to 10.0. The optimal pH for ZpaCPR was 5.5, and an increase in pH from 5.5 to 10.0 decreased the activity. In contrast, AmeLPLDH adapted to a much wider pH range (Figure 3b). Various metal ions were investigated for their effect on enzyme activity, and only Ca^2+^ slightly enhanced the enzyme activity of AmeLPLDH. None of the tested metal ions had a beneficial effect on the enzyme activity of ZpaCPR (Figure 4a). The use of organic solvents may have a profound effect on the activity of AmeLPLDH and ZpaCPR; thus, various readily available and cost-effective organic solvents (chloroform, ethyl acetate, isopropanol, acetone, octanol, and DMSO) were tested in this regard. Except for acetone, the tested organic solvents inhibited enzyme activity; octanol was the most harmful (Figure 4b). The kinetic parameters were determined, and the k_cat_/K_m_ values of AmeLPLDH and ZpaCPR were 63.85 and 11.60 mM^−1^s^−1^, respectively (Table 1). Overall, the difference in enzymatic properties between AmeLPLDH and ZpaCPR indicated that it was necessary to subsequently orchestrate their catalytic performance with BsGDH.

### 2.3. Advanced Engineering of Biocatalyst Co-Expressing AmeLPLDH, ZpaCPR, and BsGDH

**Figure 5:**
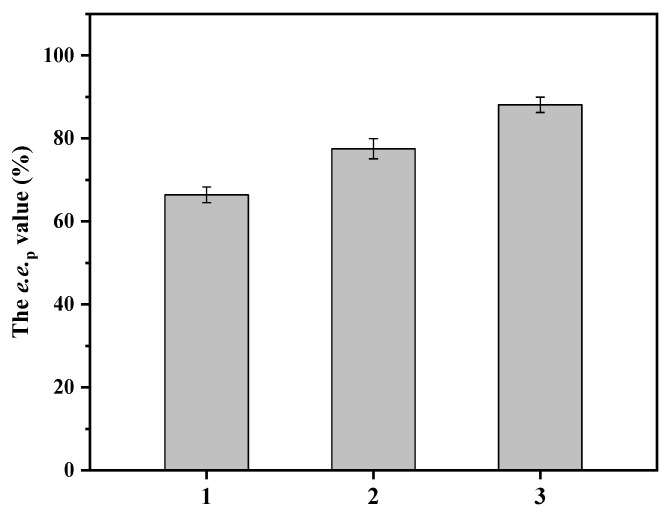
Catalytic performance of whole-cell biocatalysts based on different methods of construction. 1: *E. coli* strain BL21 (DE3)/pET28a-*AmeLPLDH*/pACYCDuet-1-*ZpaCPR-BsGDH*; 2: *E. coli* strain BL21 (DE3)/pET28a-*AmeLPLDH*/pACYCDuet-1-*ZpaCPR-(GSG)-BsGDH*; 3: *E. coli* strain BL21 (DE3)/pACYCDuet-1-*AmeLPLDH*/pET28a-*ZpaCPR-(GSG)-BsGDH*. Reaction mixture (10 mL) consisted of 200 g/L wet cells, 1 M dl-pantolactone, 1.5 M glucose, and 200 mM PBS buffer (pH 7.0). Constant pH was maintained through titration with 1 M NaOH. Reaction was run at 30 °C and 600 rpm for 24 h. Data represent mean values ± SD from three independent experiments.

In cases of multi-enzymatic co-expression, fusion between two or more enzymes could outperform combinations of individual enzymes for enhanced catalytic activity [24,25,26]. With the aim of facilitating coenzyme regeneration, the enzymes ZpaCPR and BsGDH were ligated with the linker GSG, resulting in *E. coli* strain BL21 (DE3)/pET28a-*AmeLPLDH*/pACYCDuet-1-*ZpaCPR-(GSG)-BsGDH*. Indeed, introducing the fusion protein ZpaCPR-(GSG)-BsGDH increased the *e.e.*_p_ value to 77.5%, indicating increased catalytic efficiency. Although the pET28a and pACYCDuet-1 vectors were compatible, their vector types might have affected the expression level of each gene product. The genes encoding AmeLPLDH and ZpaCPR-GSG-BsGDH exchanged vector type, resulting in *E. coli* strain BL21 (DE3)/pACYCDuet-1-*AmeLPLDH*/pET28a-*ZpaCRR-(GSG)-BsGDH*. The newly constructed whole-cell biocatalyst further increased the *e.e.*_p_ value to 88.1% (Figure 5). Thus, *E. coli* strain BL21 (DE3)/pACYCDuet-1-*AmeLPLDH*/pET28a-*ZpaCPR-(GSG)-BsGDH* was used for subsequent deracemization of dl-pantolactone at high concentrations.

### 2.4. Deracemization of dl-Pantolactone at High Concentrations

The enzymes AmeLPLDH, ZpaCPR, and BsGDH have different catalytic properties, and the deracemization process must be optimized to achieve adequate productivity and product concentrations [28]. AmeLPLDH, ZpaCPR, and BsGDH require FMN or NADPH as a coenzyme. However, neither FMN nor NADPH was exogenously supplied to the reaction mixture, so that the developed deracemization process would be cost-effective.

**Figure 6:**
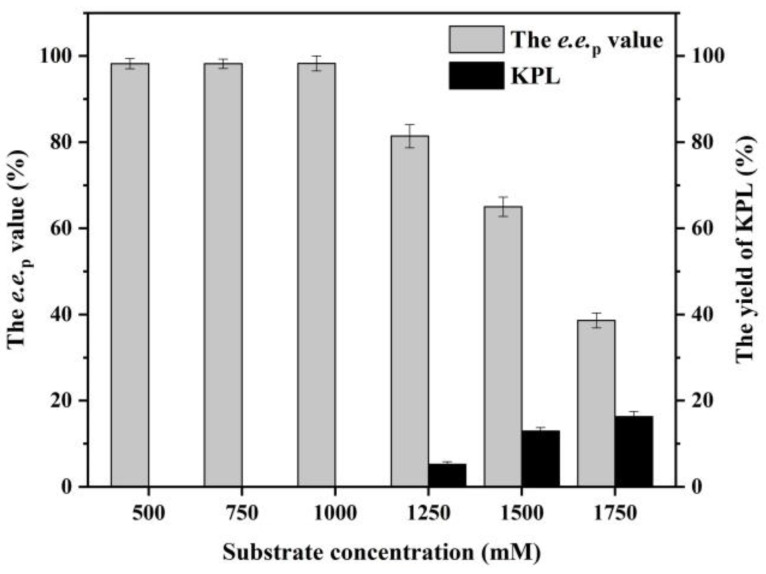
Influence of substrate concentration on three-enzyme cascade catalytic system. Reaction mixture (10 mL) consisted of 200 g/L wet cells co-expressing three enzymes, 500–1750 mM dl-pantolactone, glucose/substrate ratio of 2:1 and 200 mM PBS buffer (pH 6.0). Constant pH was maintained through titration with 1 M NaOH. Reaction was run at 30 °C and 400 rpm for 24 h. Data represent mean values ± SD from three independent experiments.

**Figure 7:**
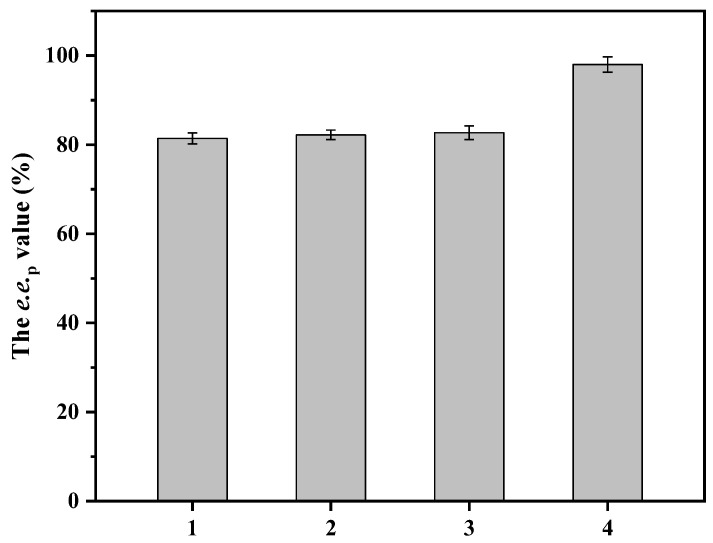
Comparison of catalytic performance with added AmeLPLDH, ZpaCPR and BsGDH after 24 h. 1: Control without enzyme supplementation; 2: supplementation with ZpaCPR; 3: supplementation with AmeLPLDH; 4: supplementation with BsGDH. Initial reaction mixture (10 mL) consisted of 200 g/L wet cells, 1.25 M dl-pantolactone, 2.5 M glucose, and 200 mM PBS buffer (pH 7.0). Constant pH was maintained through titration with 1 M NaOH. Reaction was run at 30 °C and 400 rpm for 36 h. Data represent mean values ± SD from three independent experiments.

To achieve synergistic effects, factors including temperature, pH, agitation, glucose concentration, and substrate loading were investigated. The substrate concentration was set as 1000 mM and the pH was kept constant through titration with 1 M NaOH. The optimal temperature was 30 °C, and the *e.e.*_p_ value remained >30% between 20 and 40 °C (Figure S7a). The effect of pH on catalytic efficiency was determined within the range of 5.0–10.0 (Figure S7b). The optimal pH was 6.0, and the *e.e.*_p_ value decreased as the pH rose to 10.0, indicating that the enzymatic stability was more adapted to a weak acidic environment. Agitation was optimized to 400 rpm, although the *e.e*._p_ value remained >80% between 300 and 600 rpm (Figure S7c). As a co-substrate for coenzyme regeneration, glucose in various concentrations was used to test the effect on catalytic efficiency. When no glucose was added, the *e.e*._p_ value was 24.2%, suggesting the presence of an intracellular proton supply. The *e.e.*_p_ value rose when the glucose concentration increased from 0 to 2000 mM, and concentrations higher than 2000 mM caused no further improvement. Thus, the ratio of glucose to substrate was set as 2:1. Under optimized conditions, substrate loading was increased stepwise from 500 to 1750 mM in order to investigate the effect on catalytic efficiency (Figure 6). When the substrate concentration was not greater than 1000 mM, the *e.e.*_p_ value remained at >98%. When the substrate concentration was 1250 mM, the yield of both l-pantolactone and ketopantolactone began to increase, resulting in a lower *e.e.*_p_ value (81.4%). When the substrate concentration was 1750 mM, the yield of l-pantolactone and ketopantolactone was 14.4 and 16.3%, respectively, indicating that the catalytic efficiency of the enzymes was insufficient after 24 h reaction. To identify the rate-limiting enzyme, *E. coli* cells expressing AmeLPLDH, ZpaCPR, or BsGDH were added to the reaction mixture (Figure 7). The addition of AmeLPLDH or ZpaCPR resulted in similar catalytic performance to the control, whereas supplementation with BsGDH dramatically increased the *e.e*._p_ value to 98.6%. A high proportion of d-pantolactone simplified the subsequent product separation procedure, and crude product was obtained through solvent extraction and solvent evaporation. Finally, the crude product was verified to be d-pantolactone through a combination of chiral GC, GC-MS, ^1^ H NMR, and ^13^C NMR analysis.

## 3. Materials and Methods

### 3.1. Chemicals, Genes, Plasmids, and Organisms

dl-pantolactone, ketopantolactone, and other chemicals and reagents were purchased from Shanghai Macklin Biochemical Co., Ltd. (Shanghai, China). Kits and enzymes for genetic manipulation were purchased from Takara Biomedical Technology Co., Ltd. (Beijing, China). Chromatography columns for protein purification were purchased from GE Healthcare Life Sciences (Shanghai, China). The genes encoding l-pantolactone dehydrogenase, ketopantolactone reductase and glucose dehydrogenase were codon-optimized and then synthesized by Qingke Biotechnology Co., Ltd. (Hangzhou, China). The pET28a and pACYCDuet-1 vectors were used for co-expression of l-pantolactone dehydrogenase, ketopantolactone reductase, and glucose dehydrogenase, and *E. coli* strain BL21(DE3) was used as the host. *E. coli* cells were grown routinely in LB medium at 37 °C for 12 h.

### 3.2. Construction of Toolbox through Homologous Protein-Search Analysis

The BLAST tool of the NCBI database (https://blast.ncbi.nlm.nih.gov/Blast.cgi, accessed on 20 May 2020) was used to search potential l-pantolactone dehydrogenase and ketopantolactone reductase with the NasLPLDH amino acid sequence (GenBank accession no. GAD85679.1) and *Sce*CPR1 amino acid sequence (no. NP_010159.1) as a probe. In the study, l-pantolactone dehydrogenase sequences included *Cph*LPLDH (no. CP020715.1), *Nfa*LPLDH (no. AP006618.1), *Ncy*LPLDH (no. AP026975.1), and *Ame*LPLDH (no. WP_017981470.1), and ketopantolactone reductase sequences included *Cdu*CPR (no. XP_002421511.1) and *Zpa*CPR (no. AQZ16881.1). Based on the literature [27,31,32], the selected glucose dehydrogenases included *Es*GDH (no. KM817194.1), *Bm*GDH (no. WP_033578120.1), and *Bs*GDH (no. AFQ56330.1). Clustal X 2.0 software and ESPript 3.0 were used for multiple sequence alignment.

### 3.3. Overexpression of Single LPLDH, CPR, or GDH in E. coli BL(DE3)

The genes encoding LPLDH (*Cph*LPLDH, *Nfa*LPLDH, *Ncy*LPLDH, *Nas*LPLDH, or *Ame*LPLDH), CPR (*Sce*CPR1, *Cdu*CPR, or *Zpa*CPR) and GDH (*Es*GDH, *Bm*GDH, or *Bs*GDH) were separately inserted into the *Eco*R I/*Hin*d III sites of the pET28a vector, and the resulting plasmids were individually transferred into *E. coli* BL21(DE3), yielding recombinant strains *E. coli* BL21(DE)/pET28a-*CphLPLDH*, *E. coli* BL21(DE)/pET28a-*NfaLPLDH*, *E. coli* BL21(DE)/pET28a-*NcyLPLDH*, *E. coli* BL21(DE)/pET28a-*NasLPLDH*, *E. coli* BL21(DE)/pET28a-*AmeLPLDH*, *E. coli* BL21(DE)/pET28a-*SceCRP1*, *E. coli* BL21(DE)/pET28a-*CduCPR*, *E. coli* BL21(DE)/pET28a-*ZpaCPR*, *E. coli* BL21(DE)/pET28a-*EsGDH*, *E. coli* BL21(DE)/pET28a-*BmGDH*, and *E. coli* BL21(DE)/pET28a-*BsGDH*. The recombinant strains were initially grown at 37 °C in LB medium containing 50 µg/mL kanamycin. When the OD_600_ value reached 0.6, 0.2 mM isopropyl-β-D-thiolactopyranoside (IPTG) was added to initiate induction at 24 °C for 14 h. After induction, the cells were harvested by centrifugation at 4 °C for 10 min.

### 3.4. Screening of LPLDH, CPR, and GDH Co-Expressed in E. coli BL21 (DE3)

*E. coli* BL21 (DE3)/pACYCDuet-1-*SceCPR1-EsGDH* was successfully constructed, as described previously [8]. The pET28a recombinant plasmid with the gene encoding l-pantolactone dehydrogenase was transferred into *E. coli* BL21(DE3) competent cells with the recombinant plasmid pACYCDuet-1-*SceCPR1-EsGDH*, resulting in the strains co-expressing different LPLDH, *Sce*CPR1 and *Es*GDH.

The genes encoding *Cdu*CPR and *Zpa*CPR were amplified from the plasmids pET28a-*CduCPR* and pET28a-*ZpaCPR*, respectively. The PCR program consisted of the following steps: 5 min at 98 °C, 30 cycles of 98 °C (10 s), 59 °C (15 s), and 72 °C (30 s), with a final extension at 72 °C for 5 min. The linear pACYCDuet-1-*EsGDH* fragment was amplified from the plasmid pACYCDuet-1-*SceCPR1-EsGDH* by inverse PCR. The corresponding PCR program was as follows: 5 min at 98 °C, 30 cycles of 98 °C (10 s), 58 °C (15 s), and 72 °C (90 s), with a final extension at 72 °C for 5 min. According to the instructions for the ClonExpress^®^ II One Step Cloning Kit, the genes encoding *Cdu*CPR and *Zpa*CPR were ligated with linear pACYCDuet-1-*EsGDH* fragments via Exnase II-derived homologous recombination to form recombinant plasmids pACYCDuet-1-*CduCPR-EsGDH* and pACYCDuet-1-*ZpaCPR-EsGDH*. Recombinant plasmids pACYCDuet-1-*SceCPR1-EsGDH*, pACYCDuet-1-*CduCPR-EsGDH*, and pACYCDuet-1-*ZpaCPR-EsGDH* were transferred into *E. coli* BL21(DE3) competent cells with recombinant plasmid pET28a-*AmeLPLDH*, resulting in the strains co-expressing *Ame*LPLDH, different CPR, and *Es*GDH.

Similarly, the gene encoding *Es*GDH on the plasmid pACYCDuet-1-*ZpaCPR-EsGDH* was replaced with the gene encoding *Bm*GDH or *Bs*GDH, yielding the recombinant plasmids pACYCDuet-1-*ZpaCPR-BmGDH* and pACYCDuet-1-*ZpaCPR-BsGDH*. The recombinant plasmids pACYCDuet-1-*ZpaCPR-EsGDH*, pACYCDuet-1-*ZpaCPR-BmGDH*, and pACYCDuet-1-*ZpaCPR-BsGDH* were transferred into *E. coli* BL21(DE3) competent cells with the recombinant plasmid pET28a-*AmeLPLDH*, resulting in the strains co-expressing *Ame*LPLDH, *Zpa*CPR, and different GDH. The primers for the co-expression of LPLDH, CPR, and GDH are listed in Table S1.

For each recombinant strain co-expressing LPLDH, CPR, and GDH, single colonies were picked and transferred to 50 mL tubes containing 100 μg/mL kanamycin and 100 μg/mL chloramphenicol in LB medium. The cultures were incubated at 37 °C and 200 rpm for 12 h. When the OD_600_ reached 0.6–0.8, 0.2 mM IPTG was added to initiate induction at 24 °C and 150 rpm for 14 h. Cells were washed twice using 50 mM Tris–HCl buffer (pH 8.0), then harvested by centrifuging at 8000 × *g* at 4 °C for 10 min. A 12% (*w*/*v*) SDS-PAGE was used to visualize the co-expression of LPLDH, CPR, and GDH [33].

To select LPLDH, CPR, and GDH, recombinant *E. coli* cells as whole-cell biocatalyst were used to catalyze the deracemization of dl-pantolactone into d-pantolactone. The reaction mixture (10 mL) contained 1.0 M dl-pantolactone, 1.5 M d-glucose, 200 g/L wet cells, and 200 mM PBS buffer (pH 7.0). The reaction was conducted at 30 °C and 600 rpm for 24 h, and was terminated by adding an equal volume of 3 M HCl. After that, the mixture was centrifuged and the supernatant was extracted with 5 equivalent volumes of ethyl acetate. The organic phase was harvested by centrifugation at 8000× *g* for 10 min at room temperature and subsequently dehydrated with anhydrous sodium sulfate. Finally, 200 µL of the resulting dehydrated sample was subjected to GC analysis, as described in Section 3.10.

### 3.5. Purification of AmeLPLDH and ZpaCPR

Cells expressing *Ame*LPLDH and *Zpa*CPR were resuspended in 50 mM Tris-HCl buffer (pH 8.0) at a concentration of 50 g/L, then sonicated for cell disruption. Brij 35 was added to the *Ame*LPLDH cell homogenate to give a final concentration of 1%. After gentle stirring for 2.5 h, the cell debris was removed by centrifugation. The cell-free extracts of *Ame*LPLDH and *Zpa*CPR were then analyzed by Ni-NTA chelating affinity chromatography. Unbound proteins were washed off by applying a binding buffer (5 mM imidazole and 300 mM NaCl dissolved in 50 mM Tris-HCl, pH 8.0). *Ame*LPLDH was eluted out by applying an elution buffer (300 mM imidazole and 300 mM NaCl dissolved in 50 mM Tris-HCl, pH 8.0), and *Zpa*CPR was eluted out by applying an elution buffer (200 mM imidazole and 300 mM NaCl dissolved in 50 mM Tris-HCl, pH 8.0). The purity of the purified *Ame*LPLDH and *Zpa*CPR was verified using SDS-PAGE. The purified *Ame*LPLDH and *Zpa*CPR enzymes were desalted with 50 mM Tris-HCl buffer (pH 8.0) and stored at −20 °C for further investigation.

### 3.6. Activity Assay of AmeLPLDH and ZpaCPR

The activity of *Ame*LPLDH was measured at 30 °C by monitoring changes in absorbance at 450 nm. The 1 mL assay mixture of *Ame*LPLDH consisted of 1 µg purified enzyme, 10 mM l-pantolactone, 0.15 mM FMN, and 200 mM PBS buffer (pH 8.0). The enzyme assays began with the addition of the coenzyme FMN. One unit of activity represents the reduction of 1 µmol FMN per minute. The pure enzyme activity of *Zpa*CPR was measured at 45 °C by monitoring changes in absorbance at 340 nm. The 1 mL assay mixture of *Zpa*CPR consisted of 1 µg purified enzyme, 10 mM ketopantolactone, 0.15 mM NADPH, and 200 mM PBS buffer (pH 5.5). The enzyme assay began with the addition of the coenzyme NADPH. One unit of activity represented the oxidation of 1 µmol NADPH per minute. The BCA method was used to determine the protein concentration of all samples, with bovine serum albumin used as the standard protein. All enzyme assays were performed in triplicate [34].

In the characterization of *Ame*LPLDH and *Zpa*CPR, the effects of temperature and pH on the activity were investigated at 20–50 °C and pH 5.0–10.0. The metal ions (Cu^2+^, Ni^2+^, Li^+^, Co^2+^, Na^+^, Ag^+^, Al^3+^, Ca^2+^, Mn^2+^, Zn^2+^) and organic solvents (chloroform, ethyl acetate, isopropanol, acetone, octanol, DMSO) were individually added to the reaction mixture to determine their effects on the activity. In the determination of kinetic parameters, the tested substrate concentrations included 1, 2, 4, 6, 8, 10, 20, 30, and 40 mM. According to Michaelis–Menten kinetics, the parameters *K*_m_ and *V*_max_ were calculated through curve fitting using Origin Pro software (version 8.5).

### 3.7. Co-Expression of AmeLPLDH and Fusion Enzyme ZpaCPR-(GSG)-BsGDH

The fusion genes encoding *Zpa*CPR and *Bs*GDH were constructed by multiple overlap extension PCR. To assemble the *Zpa*CPR-(linker)-*Bs*GDH fusion gene, the stop codon of the *Zpa*CPR gene was removed and the linker GSG was introduced between the open reading frames of the genes encoding *Zpa*CPR and *Bs*GDH via two rounds of PCR. The first round of PCR introduced the linker GSG into the *Zpa*CPR gene using one pair of primers (Table S2). Simultaneously, the complementary linker GSG was introduced into the *Bs*GDH gene using the other pair of primers (Table S2). Each PCR product was purified and served as a template in the second round of PCR. The PCR program was as follows: 3 min at 98 °C, 30 cycles at 98 °C (10 s), 58 °C (15 s), and 72 °C (30 s), and a final 10 min extension at 72 °C. The PCR products of the *Zpa*CPR and *Bs*GDH genes were joined by overlap extension PCR. The PCR program was as follows: 3 min at 98 °C, 30 cycles at 98 °C (10 s), 58 °C (15 s), and 72 °C (40 s), and a final 5 min extension at 72 °C. The purified PCR products were ligated into a pACYCDuet-1 vector. The fusion gene was confirmed by sequencing. Finally, the fusion gene was ligated into pACYCDuet-1 between *Eco*R I and *Hin*d III sites, yielding pACYCDuet-1-*ZpaCPR-(GSG)-BsGDH*. Using the construction of pACYCDuet-1-*ZpaCPR-(GSG)-BsGDH* plasmid as an example, pET28a-*ZpaCPR-(GSG)-BsGDH* was also constructed (Figure S8). Plasmids pACYCDuet-1-*ZpaCPR-(GSG)-BsGDH* and pET28a-*ZpaCPR-(GSG)-BsGDH* were transformed into competent cells with pET28a-*AmeLPLDH* and pACYCDuet-1-*AmeLPLDH*, respectively, resulting in *E. coli* strains BL21 (DE3)/pET28a-*AmeLPLDH*/pACYCDuet-1-*ZpaCPR-(GSG)-BsGDH* and BL21 (DE3)/pET28a-*ZpaCPR-(GSG)-BsGD*H/pACYCDuet-1-*AmeLPLDH*. Following the procedure in Section 3.4, the cells were induced and harvested; then, the catalytic performance in the deracemization of dl-pantolactone was determined.

### 3.8. Optimization of Multi-Enzymatic Deracemization of dl-Pantolactone

*E. coli* strain BL21 (DE3)/pACYCDuet-1-*AmeLPLDH*/pET28a-*ZpaCPR-(GSG)-BsGDH* was used as whole-cell catalyst. The initial reaction mixture (10 mL) contained 1 M dl-pantolactone, 200 g/L wet cells, 1.5 M glucose, and 200 mM PBS buffer (pH 6.0). The reaction was carried out at 30 °C and 600 rpm for 24 h. The pH was kept constant during the reaction by stream addition of 1 M NaOH solution.

The optimal conditions for the process were investigated using a single factor method. Temperature varied from 20 to 40 °C, and pH values ranged from 5.0 to 10.0. Agitation between 0 and 600 rpm and the concentration of glucose as co-substrate within the range of 0 to 2500 mm were tested. In addition, the effects of substrate concentration (500 to 1750 mM) on the deracemization of dl-pantolactone were also determined. After 24 h reaction, the samples were treated as described in Section 3.4, then subjected to GC analysis as described in Section 3.10.

### 3.9. Deracemization of 1.25 mM dl-Pantolactone through Supplementation with BsGDH

After optimization, the reaction mixture (10 mL) contained 1.25 M dl-pantolactone, 2.5 M glucose, 200 g/L wet *E. coli* BL21 (DE3)/pACYCDuet-1-*AmeLPLDH*/pET28a-*ZpaCPR-(GSG)-BsGDH* cells, and 50 mM PBS buffer (pH 6.0). After 24 h reaction at 30 °C and 400 rpm, 100 g/L wet *E. coli* BL21 (DE3)/pET28a-*BsGDH* cells were added to the reaction mixture. Then, the reaction proceeded at 30 °C and 400 rpm for another 12 h. For the whole reaction, pH was kept constant using an auto-titration system. When the reaction was terminated at 36 h, the samples were treated as described in Section 3.4, then subjected to GC analysis as described in Section 3.10.

### 3.10. GC, GC-MS and NMR Analysis

The details of the method for analyzing l-pantolactone, d-pantolactone, and ketopantolactone by gas chromatography (Agilent 7890A, Agilent Technologies, Inc., Santa Clara, CA, USA) are as follows: detector: FID; chiral capillary column: BGB-174 (30 m × 250 µm × 0.25 µm; BGB Analytik, Böckten, Switzerland); carrier gas: N_2_; flow rate: 30.0 mL/min; split ratio: 30:1; injection volume: 1 µL; injector and detector temperature: 250 °C [8]. The column temperature was kept constant at 175 °C for 10 min. The retention times for d-pantolactone, l-pantolactone, and ketopantolactone were 6.19, 6.51, and 6.78 min, respectively (Figure S9).

Upon completion of the catalytic reaction, the reaction mixture was treated with ethyl acetate; then, the product in the resulting organic phase was collected through evaporation of the solvent. The resulting crude product was validated by GC-MS (Agilent 7890A/5975C, Agilent Technologies Inc., Santa Clara, CA, USA) using previously reported parameters (Figure S10) [8]. The crude product was dissolved in CDCl_3_ for NMR analysis (Avance NEO, Bruker, Switzerland) to further verify the remaining product, d-pantolactone. The NMR spectroscopy was operated at 600 MHz for ^1^H and 151 MHz for ^13^C detection (Figure S11).

## 4. Conclusions

In summary, a toolbox consisting of five LPLDHs, three CPRs, and three GDHs was developed for carrying out multi-enzymatic deracemization of dl-pantolactone. By screening catalytic activity, the enzymes *Ame*LPLDH, *Zpa*CPR, and *Bs*GDH were selected, and the newly mined enzymes *Ame*LPLDH and *Zpa*CPR were purified and then characterized. The *k*_cat_/*K*_m_ values of *Ame*LPLDH and *Zpa*CPR were 63.85 and 11.60 mM^−1^s^−1^, respectively. Through genetic fusion and vector selection, *E. coli* strain BL21(DE3)/pACYCDuet-1-*AmeLPLDH*/pET28a-*ZpaCPR-(GSG)-BsGDH* was obtained and used as whole-cell catalyst, providing a competitive catalytic process to synthesize d-pantolactone with high optical purity (>98% *e.e*._p_). Under the optimized conditions, the process enabled efficient deracemization of 1 M dl-pantolactone at 24 h, and supplementation with *Bs*GDH further yielded nearly complete deracemization of 1.25 M dl-pantolactone to d-pantolactone after 36 h.

## Figures and Tables

**Table 1:** Kinetic parameters of *Ame*LPLDH and *Zpa*CPR.

Parameter	*Ame*LPLDH	*Zpa*CPR
*V*_max_ (U/mg)	52.55 ± 0.42	72.14 ± 3.16
*K*_m_ (mM)	0.57 ± 0.04	3.65 ± 0.58
*k*_cat_ (s^−1^)	36.41 ± 0.29	42.22 ± 1.85
*k*_cat_/*K*_m_ (s^−1^ mM^−1^)	63.85 ± 0.51	11.60 ± 0.50

Note: *AmeL*PLDH assay mixture (1 mL) consisted of 1 μg purified enzyme, 1~40 mM l-pantolactone, 0.15 mM FMN, and 200 mM PBS buffer (pH 8.0). Activity was measured in triplicate at 30 °C by monitoring changes in absorbance at 450 nm. *Zpa*CPR assay mixture consisted of 1 μg purified enzyme, 1~40 mM ketopantolactone, 0.15 mM NADPH and 200 mM PBS buffer (pH 5.5). Activity was measured in triplicate at 45 °C by monitoring changes in absorbance at 340 nm. Data represent mean values ± SD from three independent experiments.

## Data Availability

The data presented in this study are available in the Supplementary Materials.

## Supplementary Materials

The following supporting information can be downloaded at https://www.mdpi.com/article/10.3390/molecules28145308/s1: Table S1: Primers used for co-expression of LPLDH, CPR and GDH; Table S2: Primers used for genetic fusion of *Bs*GDH and *Zpa*CPR genes; Figure S1: SDS-PAGE analysis of l-pantolactone dehydrogenases (LPLDHs); Figure S2: SDS-PAGE analysis of co-expression of LPLDH, *Sce*CPR1 and *Es*GDH; Figure S3: SDS-PAGE analysis of co-expression of *Ame*LPLDH, different CPRs and *Es*GDH; Figure S4: SDS-PAGE analysis of co-expression of *Ame*LPLDH, *Zpa*CPR and different GDHs; Figure S5: SDS-PAGE (12%) analysis of (a) *Zpa*AR and (b) AmeLPLDH; Figure S6: SDS-PAGE analysis of *E. coli* cells co-expressing *Ame*LPLDH and fusion enzyme *Zpa*CPR-(GSG)-*Bs*GDH; Figure S7: Effects of (a) temperature, (b) pH, (c) agitation and (d) glucose concentration on catalytic performance; Figure S8: Schematic diagram of *Zpa*CPR-(GSG)-*Bs*GDH fusion enzyme construction; Figure S9: GC chromatogram of standards of substrate and product; Figure S10: GC-MS chromgatogram of d-pantolactone; Figure S11: (a) ^1^H NMR and (b) ^13^C NMR analysis of product.

## Sample Availability

Samples of the compounds are not available from the authors upon request.
