# Supplementary material for: Multi-Enzymatic Cascade for Efficient Deracemization of dl-Pantolactone into d-Pantolactone
Source: Molecules. 2023 Jul 10;28(14):5308. doi: 10.3390/molecules28145308 (PMC10384591; doi:10.3390/molecules28145308)
Supplement: Supplementary file 1 [file molecules-28-05308-s001.zip › molecules-2436955-supplementary.pdf]

Supporting Information

Multi-enzymatic Cascade for Efficient Deracemization of DL-Pantolactone to D-Pantolactone

Lijun Jin [1], Xun Liu [1], Tairan Wang [1], Yi Wang [1], Xueting Zhou [1], Wangwei Mao [1], Yinjun Zhang [1], Zhao Wang [1], Jie Sun [1,*] and Xiangxian Ying [1,*]

[1] Key Laboratory of Bioorganic Synthesis of Zhejiang Province, College of Biotechnology and Bioengineering, Zhejiang University of Technology, Hangzhou 310014, China; jinlijun130@163.com (L.J.); lx0925124X@163.com (X.L.); 13591715817@163.com (T.W.); huzaiziy@163.com (Y.W.); sadie598851321@163.com (X.Z.); yingxx22@foxmail.com (W.M.); zhangyj@zjut.edu.cn (Y.Z.); hzwangzhao@163.com (Z.W.)

* Correspondence: jsun@zjut.edu.cn (J.S.); yingxx@zjut.edu.cn (X.Y.)

Table of Contents

Supplementary tables 2

Table S1 The primers used for co-expression of LPLDH, CPR and GDH 2

Table S2 The primers used for genetic fusion of *Bs*GDH and *Zpa*CPR genes 3

Supplementary figures 4

Figure S1 SDS-PAGE analysis of different L-pantolactone dehydrogenases (LPLDHs). 4

Figure S2 SDS-PAGE analysis of co-expression of LPLDH, *Sce*CPR1 and *Es*GDH. 5

Figure S3 SDS-PAGE analysis of co-expression of *Ame*LPLDH, different CPRs and *Es*GDH. 6

Figure S4 SDS-PAGE analysis of co-expression of *Ame*LPLDH, *Zpa*CPR and different GDHs. 7

Figure S5 SDS-PAGE (12%) analysis of *Zpa*CPR (a) and *Ame*LPLDH (b). 8

Figure S6 SDS-PAGE analysis of *E. coli* cells co-expressingof *Ame*LPLDH and the fusion enzyme *Zpa*CPR-(GSG)-*Bs*GDH. 9

Figure S7 The effects of temperature (a), pH (b), agitation (c) and glucose concentration (d) on catalytic performance. 10

Figure S8 Schematic diagram of *Zpa*CPR-(GSG)-*Bs*GDH fusion enzyme construction 11

Figure S9 The GC chromatogram of the standards of substrate and product 12

Figure S10 GC-MS chromgatogram for D-pantolactone 13

Figure S11 The ^{1}H NMR (a) and ^{13}C NMR (b) analyses of the product 14

Supplementary tables

Table S1 The primers used for co-expression of LPLDH, CPR and GDH

Primer	Sequence
F-*Bm*GDH	5’-ATGTATAAAGATCTGGAAGGTAAA-3’
R-*Bm*GDH	5’-ACCACGACCTGCCTGAAA-3’
F-*Bs*GDH	5’-ATGTATATGTATCCGGATCTGA-3’
R-*Bs*GDH	5’-ACCACGACCCGCCTGAAAG-3’
F-*Sce*CPR1	5’-ATGGGCTCATTTCATCAGCAGTTCTTCA-3’
R-*Sce*CPR1	5’-CACTTTCTGGGCCGCATAATTATATTTGC-3’
J-F-*Bm*GDH	5’-CACAGCCAGGATCCGAATTCATGTATAAAGATCTGGAAGGTAA-3’
J-R-*Bm*GDH	5’-AATTATAACCCATCCCTGACCCACCACGACCTGCCTGAAA-3’
J-F-*Bs*GDH	5’-CCACAGCCAGGATCCGAATTCATGTATATGTATCCGGATCTGA-3’
J-R-*Bs*GDH	5’-AATTATAACCCATCCCTGACCCACCACGACCCGCCTGAAAG-3’
J-F-*Sce*CPR1	5’-ACAGCCAGGATCCGAATTCATGGGCTCATTTCATCAGCAGTTCT-3’
J-R-*Sce*CPR1	5’-AATATAATTATGCGGCCCAGAAAGTGGGGTCAGGGATGGGTTA-3’
F-*Cdu*CPR	5’-ATGACCAGCCATACCCATCCGGTG-3’
R-*Cdu*CPR	5’-CAGATCTTTAAATGCTTCATGA-3’
F-*Zpa*CPR	5’-ATGCCGAAACTGCCGGCCCCGAC-3’
R-*Zpa*CPR	5’-ACTACGAAATTGTGCAAA-3’
J-F-*Cdu*CPR	5’-CACAGCCAGGATCCGAATTCATGACCAGCCATACCCATCCGGT-3’
J-R-*Cdu*CPR	5’-AGTGGGGTCAGGGATGGGTTATAATTCAGATCTTTAAATGCTTC-3’
J-F-*Zpa*AR	5’-CCACAGCCAGGATCCGAATTCATGCCGAAACTGCCGGCCCCGA-3’
J-R-*Zpa*AR	5’-GCATTATGCGGCCGCAAGCTTTCAACTACGAAATTGTGCAAA-3’

Table S2 The primers used for genetic fusion of *Bs*GDH and *Zpa*CPR genes

Primer	Sequence
F-*Bs*GDH-R-*Zpa*CPR-pA	5'-CGTAGTGGGTCAGGGATGTATATGTATCCGGATCTGA-3'
R-*Bs*GDH-R-*Zpa*CPR-pA	5'-TCGAGTGCGGCCGCAAGCTTACTACGAAATTGTGCAAAGAATT-3'
F-*Zpa*CPR-R-*Bs*GDH-pA	5'-TCGCGGATCCGAATTCATGCCGAAACTGCCGGC-3'
R-*Zpa*CPR-R-*Bs*GDH-pA	5'-ATATACATCCCTGACCCACTACGAAATTGTGCAAAGAATTCG-3'
F-*Bs*GDH-R-*Zpa*CPR-pE	5'-TCCAGCGGGTCAGGGATGTATATGTATCCGGATCTGA-3'
R-*Bs*GDH-R-*Zpa*CPR-pE	5'-TCGAGTGCGGCCGCAAGCTTACTACGAAATTGTGCCCTAGC-3'
F-*Zpa*CPR-R-*Bs*GDH-pE	5'-TCCAGCATCCGAATTCATGCCGAAACTGCCGGC-3'
R-*Zpa*CPR-R-*Bs*GDH-pE	5'-CATCCCTGACCCACTACGAAATTGTGCAAAGAATTCGCCTAGC-3'
J-F-*Zpa*AR-R-*Bs*GDH	5'-GCGGATCCGAATTCATGCCGAAACTGCCG-3'
J-R-*Zpa*AR-R-*Bs*GDH	5'-TACATCCCTGACCCACTACGAAATTGTGCAAAGAA-3'

Supplementary figures

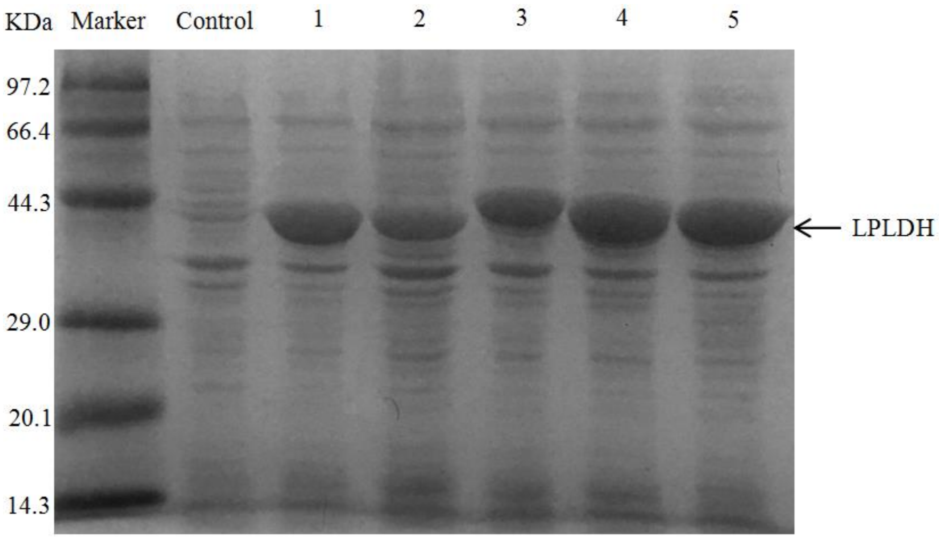

Figure S1 SDS-PAGE analysis of different L-pantolactone dehydrogenases (LPLDHs). Lane M, marker; lane 1, no induction of LPLDH; lane 2, *Ame*LPLDH (41.6 kDa); lane 3, *Cph*LPLDH (41.6 kDa); lane 4, *Nas*LPLDH (41.6 kDa); lane 5, *Nfa*LPLDH (41.6 kDa); lane 6, *Ncy*LPLDH (41.6 kDa).

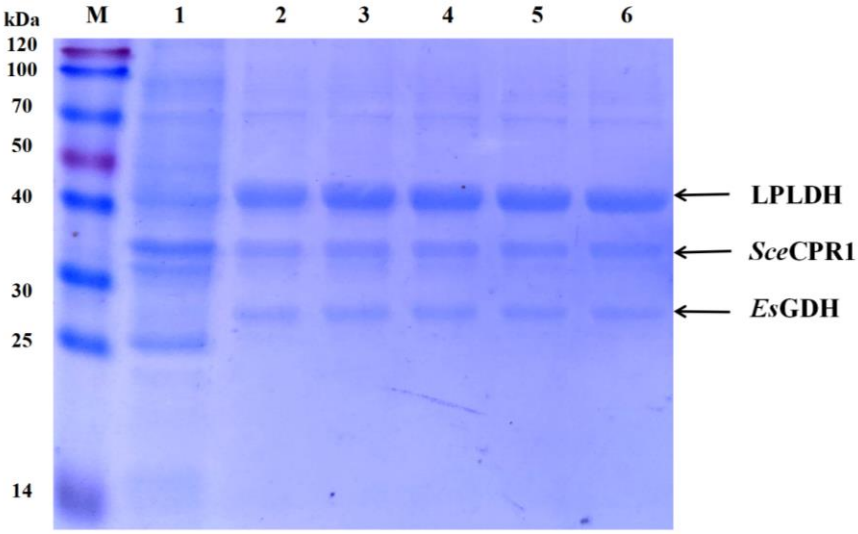

Figure S2 SDS-PAGE analysis of co-expression of LPLDH, *Sce*CPR1 and *Es*GDH. Lane M, marker; lane 1, no induction of LPLDH, *Sce*CPR1 and *Es*GDH as the control; lane 2, co-expression of *Ame*LPLDH (41.6 kDa), *Sce*CPR1 (35.6 kDa) and *Es*GDH (28.1 kDa); lane 3, co-expression of *Cph*LPLDH (41.6 kDa), *Sce*CPR1 and *Es*GDH; lane 4, co-expression of *Nas*LPLDH (41.6 kDa), *Sce*CPR1 and *Es*GDH; lane 5, co-expression of *Nfa*LPLDH (41.6 kDa), *Sce*CPR1 and *Es*GDH; lane 6, co-expression of *Ncy*LPLDH (41.6 kDa), *Sce*CPR1 and *Es*GDH.

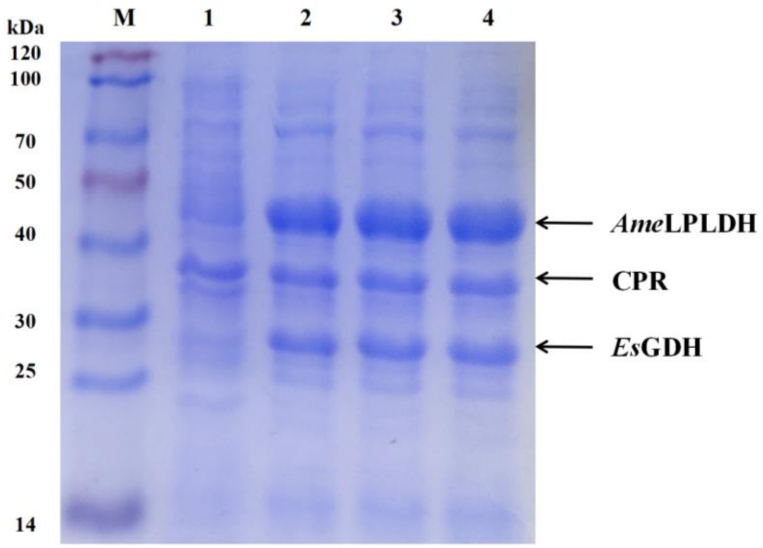

Figure S3 SDS-PAGE analysis of co-expression of *Ame*LPLDH, different CPRs and *Es*GDH. Lane M, marker; lane 1, no induction of *Ame*LPLDH, CPR and *Es*GDH as the control; lane 2, co-expression of *Ame*LPLDH (41.6 kDa), *Sce*CPR1 (35.6 kDa) and *Es*GDH (28.1 kDa); lane 3, co-expression of *Ame*LPLDH, *Cdu*CPR (35.2 kDa) and *Es*GDH; lane 4, co-expression of *Ame*LPLDH, *Zpa*CPR (35.2 kDa) and *Es*GDH.

.

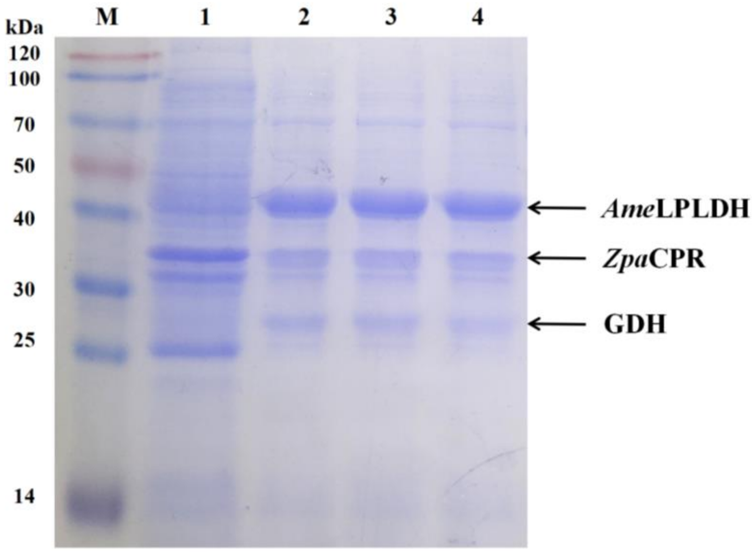

Figure S4 SDS-PAGE analysis of co-expression of *Ame*LPLDH, *Zpa*CPR and different GDHs. Lane M, marker; lane 1, no induction of *Ame*LPLDH, *Zpa*CPR and GDH as the control; lane 2, co-expression of *Ame*LPLDH (41.6 kDa), *Zpa*CPR (35.2 kDa) and *Bs*GDH (28.1 kDa); lane 3, co-expression of *Ame*LPLDH, *Zpa*CPR and *Es*GDH (28.1 kDa); lane 4, co-expression of *Ame*LPLDH, *Zpa*CPR and *Bm*GDH (28.1 kDa).

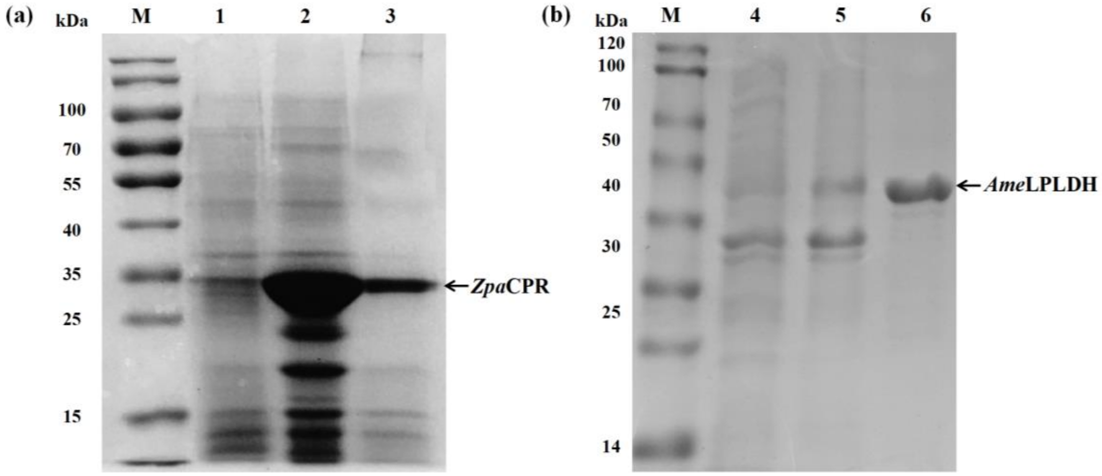

Figure S5 SDS-PAGE (12%) analysis of *Zpa*CPR (a) and *Ame*LPLDH (b). Lane M, standard molecular mass proteins; lane 1 and lane 4, the host train *E. coli* BL21(DE3); lane 2, crude *Zpa*CPR; lane 3, *Zpa*CPR after purification; lane 5, crude *Ame*LPLDH; lane 6, *Ame*LPLDH after purification.

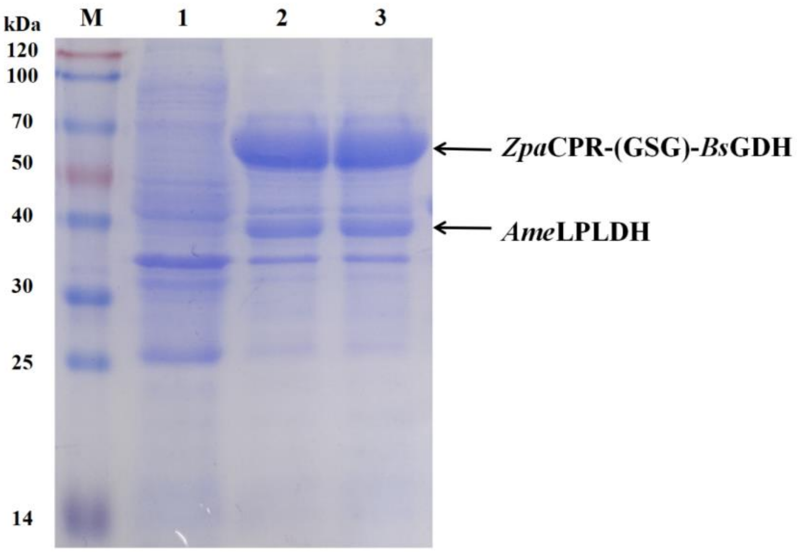

Figure S6 SDS-PAGE analysis of *E. coli* cells co-expressingof *Ame*LPLDH and the fusion enzyme *Zpa*CPR-(GSG)-*Bs*GDH. Lane M, standard molecular mass proteins; lane 1, the host train *E. coli* BL21(DE3); lane 2, the strain *E. coli* BL21 (DE3)/pET28a-*AmeLPLDH*/pACYCDuet-1-*ZpaCPR-(GSG)-BsGDH*; lane 3, the strain *E. coli* BL21 (DE3)/pACYCDuet-1-*AmeLPLDH*/pET28a-*ZpaCPR-(GSG)-BsGDH*.

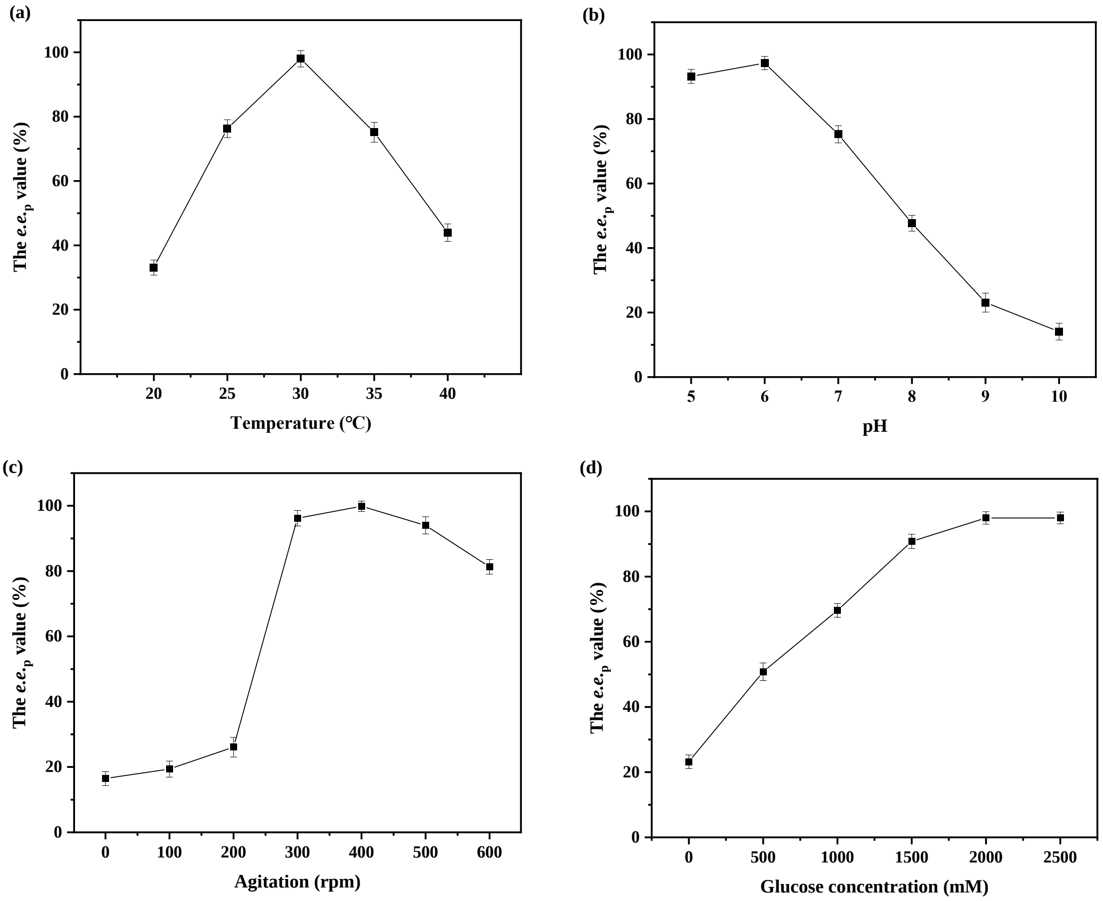

Figure S7 The effects of temperature (a), pH (b), agitation (c) and glucose concentration (d) on catalytic performance. The reaction mixture (10 mL) consisted of 200 g/L wet cells co-expressing three enzymes, 1 M DL-Pantolactone, 0-2.5 M glucose and 200 mM PBS buffer (pH 5.0-10.0). Constant pH was kept through the titration of 1 M NaOH. The reaction was run at 20-40 °C and 0-600 rpm for 24 h. Data present mean values ± SD from three independent experiments.

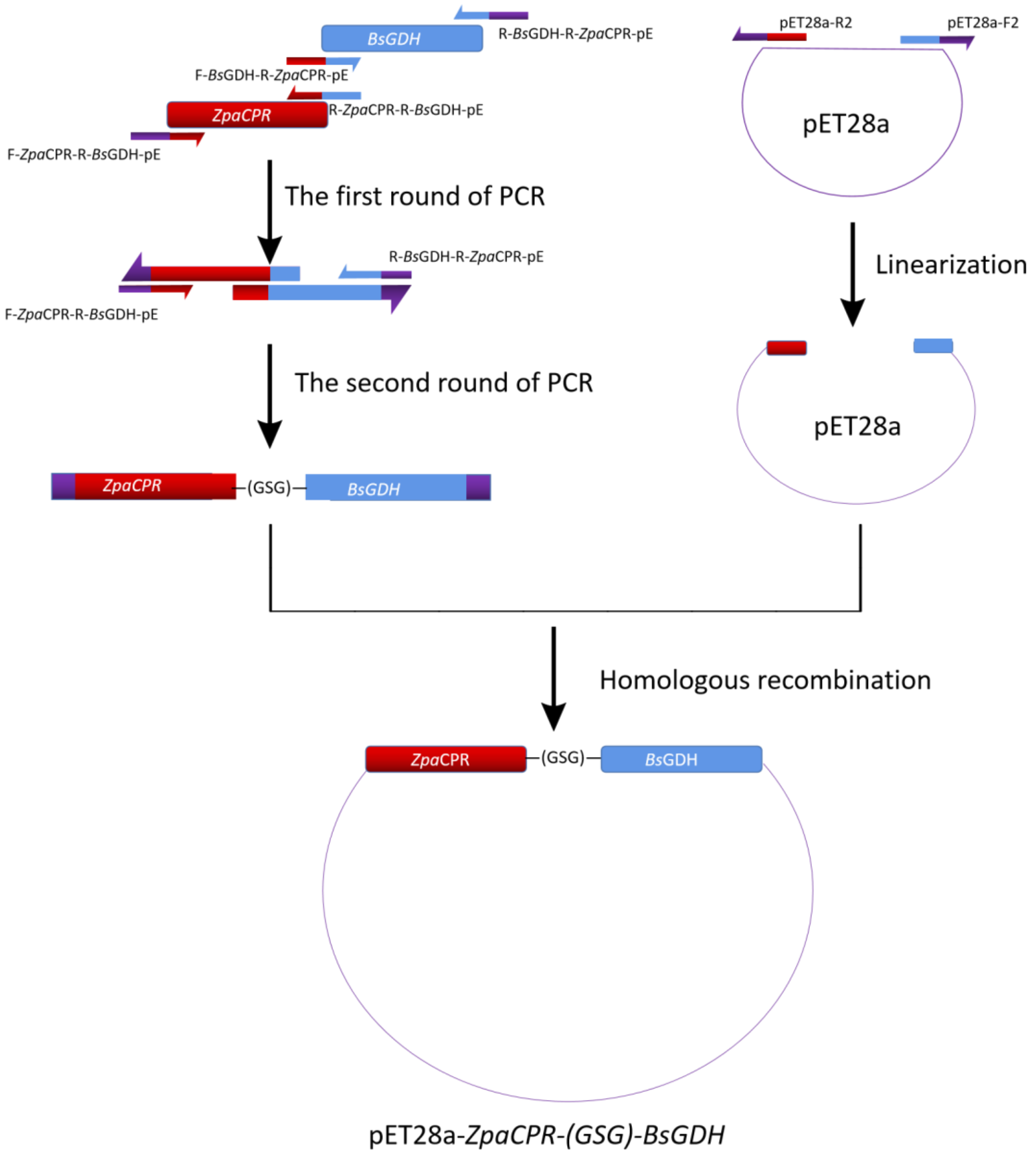

Figure S8 Schematic diagram of *Zpa*CPR-(GSG)-*Bs*GDH fusion enzyme construction.

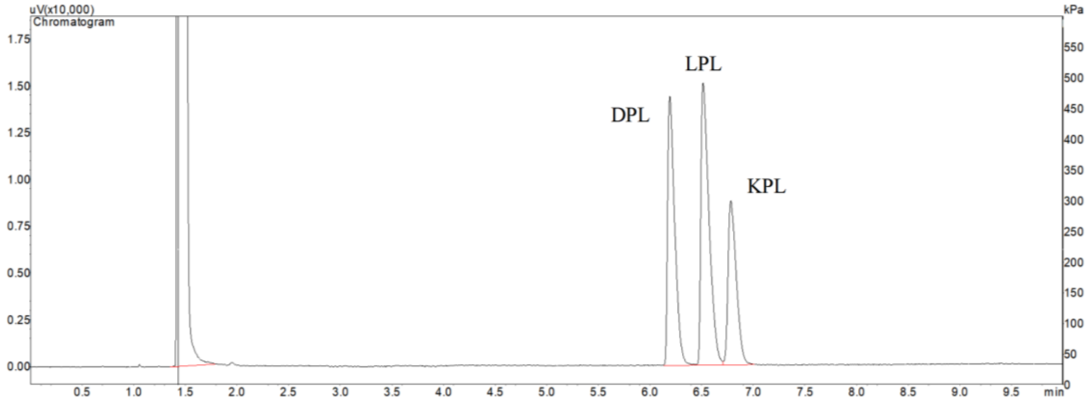

Figure S9 The GC chromatogram of the standards of substrate and product. The retention times of D-pantolactone (DPL), L-pantolactone (LPL) and ketopantolactone (KPL) were 6.192 min, 6.514 min and 6.783 min, respectively.

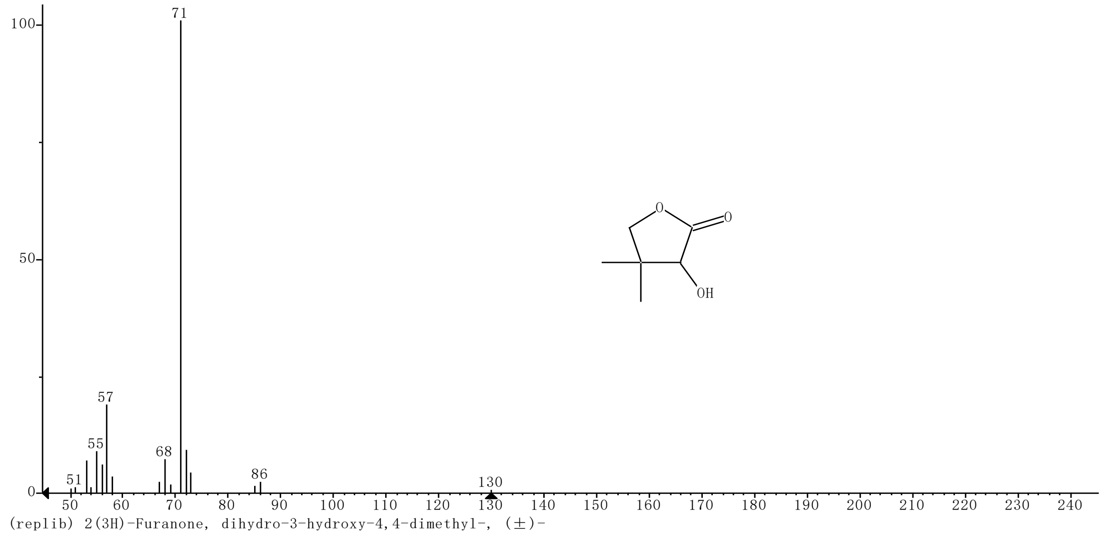

Figure S10 GC-MS chromgatogram for D-pantolactone (MW 130).

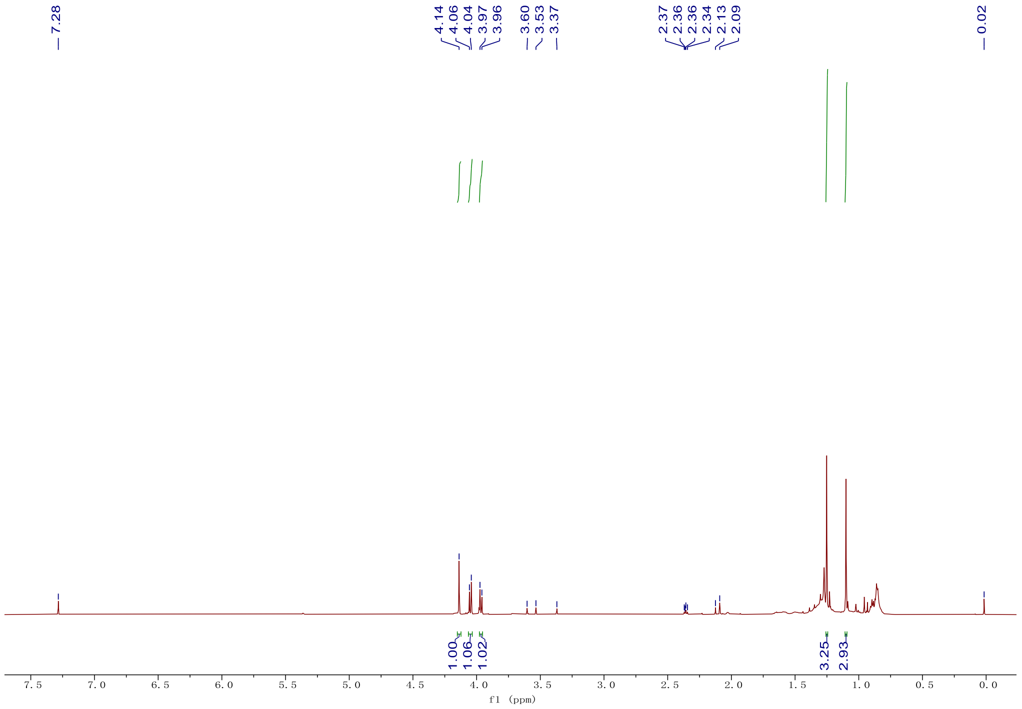

(a)

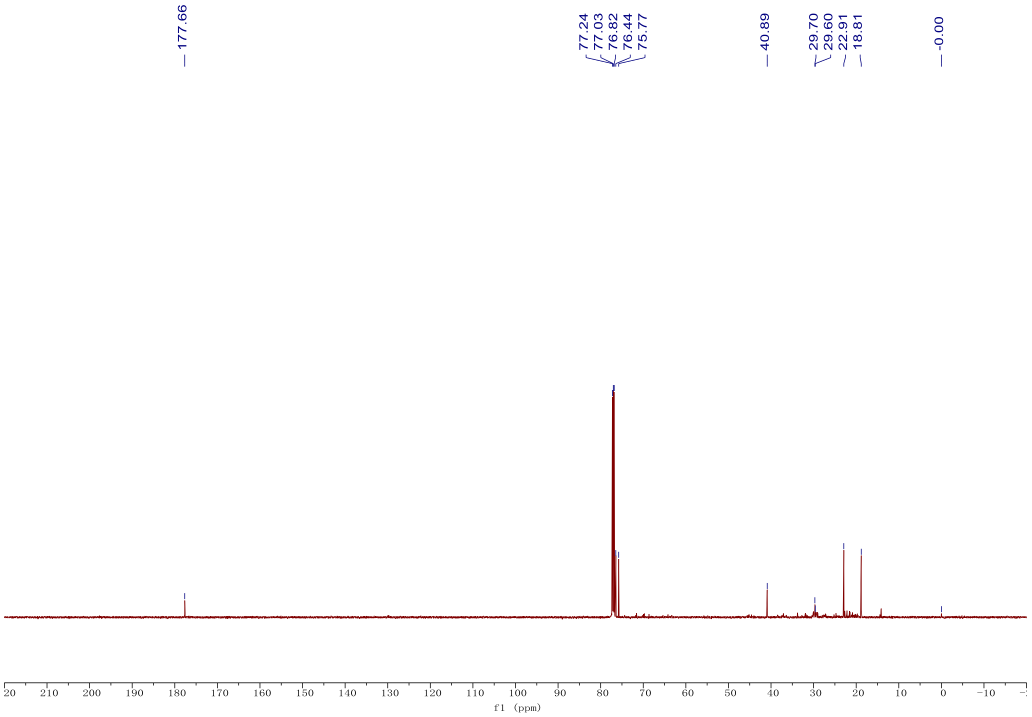

(b)

Figure S11 The ^{1}H NMR (a) and ^{13}C NMR (b) analyses of the product D-pantolactone. (a), ^{1}H NMR (600 MHz, Chloroform-*d*) δ 4.14 (s, 1H), 4.05 (d, *J* = 8.9 Hz, 1H), 3.97 (d, *J* = 9.0 Hz, 1H), 1.25 (s, 3H), 1.10 (s, 3H). (b), ^{13}C NMR (151 MHz, Chloroform-*d*) δ 177.66, 76.44, 75.77, 40.89, 22.91, 18.81.
